# Role of Nitrate Reductase in NO Production in Photosynthetic Eukaryotes

**DOI:** 10.3390/plants8030056

**Published:** 2019-03-06

**Authors:** Manuel Tejada-Jimenez, Angel Llamas, Aurora Galván, Emilio Fernández

**Affiliations:** Departamento de Bioquímica y Biología Molecular, Campus de Rabanales y Campus Internacional de Excelencia Agroalimentario (CeiA3), Edif. Severo Ochoa, Universidad de Córdoba, 14071 Córdoba, Spain; manuel.tejada@uco.es (M.T.-J.); bb2llaza@uco.es (A.L.); bb1gacea@uco.es (A.G.)

**Keywords:** nitric oxide, nitrate reductase, NOFNiR, nitrogen metabolism

## Abstract

Nitric oxide is a gaseous secondary messenger that is critical for proper cell signaling and plant survival when exposed to stress. Nitric oxide (NO) synthesis in plants, under standard phototrophic oxygenic conditions, has long been a very controversial issue. A few algal strains contain NO synthase (NOS), which appears to be absent in all other algae and land plants. The experimental data have led to the hypothesis that molybdoenzyme nitrate reductase (NR) is the main enzyme responsible for NO production in most plants. Recently, NR was found to be a necessary partner in a dual system that also includes another molybdoenzyme, which was renamed NO-forming nitrite reductase (NOFNiR). This enzyme produces NO independently of the molybdenum center of NR and depends on the NR electron transport chain from NAD(P)H to heme. Under the circumstances in which NR is not present or active, the existence of another NO-forming system that is similar to the NOS system would account for NO production and NO effects. PII protein, which senses and integrates the signals of the C–N balance in the cell, likely has an important role in organizing cell responses. Here, we critically analyze these topics.

## 1. Introduction

Nitric oxide (NO) is a gaseous secondary messenger in humans, animals, plants, fungi, and bacteria. In plants, NO is involved in important physiological processes, such as growth, development, metabolism, leaf senescence, biotic and abiotic stress, defense processes, and plant–pathogen interactions, which have been extensively reviewed [1,2,3,4,5]. In particular, in algae, such as the green alga *Chlamydomonas reinhardtii*, NO also participates in fundamental cell functions, such as the regulation of N-metabolism, N- and S-starvation stress, chloroplast biogenesis, programmed cell death, and responses to darkness, hypoxia, or salt stress [6,7,8,9,10,11].

In the last two decades, it has been clarified that NO is a signaling molecule in plant defense during plant–pathogen interactions [12,13]. Since then, different strategies have been used to understand NO biosynthesis in plant cells, and this subject has not been short of controversies [5,14], with some aspects yet to be understood. Two main pathways, reductive and oxidative, appear to explain NO synthesis in plants. One is based on the reduction of nitrite, and the other involves the oxidation of aminated molecules, such as the amino acid arginine [15].

In spite of the seminal work of Foresi and collaborators, who identified the first NO synthase (NOS) from the plant kingdom in the green alga *Ostreococus taurii* [16], the existence of a plant NOS that has the characteristics of the animal NOS has been puzzling [14,17] since no plant genome contains such a conserved gene. In fact, Jeandrof and collaborators analyzed over 1000 species of land plants and algae and found no typical NOS sequences in the 1087 sequenced transcriptomes of land plants, but they did find said sequences in 15 of the 265 algal species. Thus, it was concluded that land plants had evolved a mechanism to synthesize NO in a manner that is different from that used in animals [18]. In this review, the different biosynthetic processes of nitric oxide formation are critically analyzed, together with their physiological relevance.

## 2. Nitrite: The Substrate for Reductive NO Production

Nitrite is a product of nitrate reductase (NR)-catalyzed nitrate reduction within the nitrate assimilation pathway. Nitrogen acquisition is a fundamental process for living beings, including plants in crops, in which N is usually a limiting factor that determines crop productivity [19]. Nitrate used to be the preferred form of inorganic N that was available in soils and, thus, was used in fertilizers [20]. 

**Figure 1:**
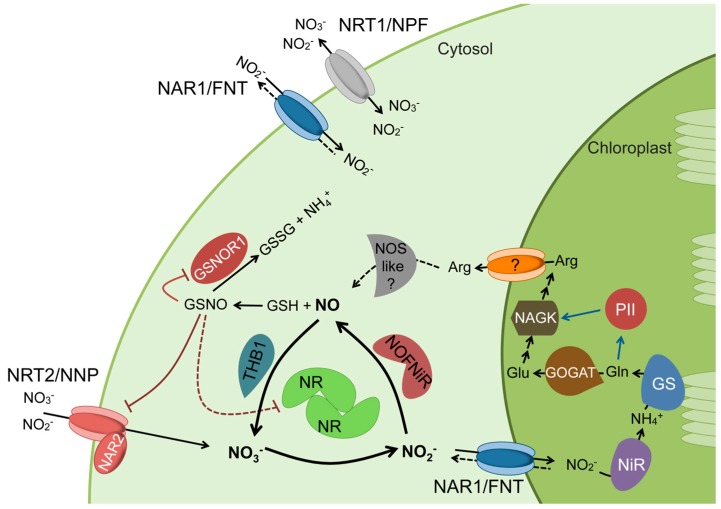
The schematic model for the coordinated regulation of Nitric Oxide (NO) synthesis and N metabolism. Blue arrows indicate activation and red lines indicate inhibition by trans-nitrosylation. Dashed lines represent hypothetical steps. The NOS-like component represents the L-Arg-dependent NOS activity reported in different plant species.

The incorporation of nitrogen from nitrate first requires its acquisition from the medium by specific transporters, which are responsible for the sensing, uptake, storage, and distribution of nitrate among plant tissues. Plant nitrate transporters belong to several families: the nitrate transporter 1/peptide transporter/nitrate peptide transporter family (NRT1/PTR/NPF), NRT2/nitrate nitrite porter (NRT2/NNP), chloride channels (CLC), slow anion channel-associated 1 homolog 3 (SLAC1/SLAH), and aluminum-activated malate transporters (ALMT). These have all been reviewed in detail [21,22,23,24,25]. In the Chlamydomonas alga, this complexity is less but still significant, reflecting the importance of this step. Here, we highlight the three families of transporter proteins found in Chlamydomonas: NRT1/NPF, NRT2, and NAR1 (Figure 1). NRT1 has been described in Arabidopsis as a dual-affinity nitrate/nitrite transporter, and NRT2 (with the accessory protein NAR2) mediates the high-affinity transport (HAT) of nitrate and nitrite. 

Nitrite in the cytosol, either produced from nitrate or absorbed from the medium, has to be transported to the chloroplast by a HAT system. In Chlamydomonas, HAT of nitrite is facilitated by NAR1, which belongs to the FNT family and is absent in land plants. In higher plants, nitrite transport to chloroplasts is typically mediated by members of the CLC family [26], and it is exported from the chloroplast by a transporter from the NRT1 family, as is found in cucumber, *Cucumis sativus* [27,28]. Regardless, nitrite concentrations in the cytosol are maintained at very low levels (micromolar range) [29] to prevent nitrite toxicity in the cell [30]. Once in the chloroplast, nitrite is reduced to ammonium in a reaction catalyzed by nitrite reductase (NiR). All of these genes (NRT2/NAR2/NR/NAR1/NiR) in Chlamydomonas are controlled by the master regulatory gene for nitrate assimilation: *NIT2* [31]. Orthologous regulatory genes in land plants—*NLP* genes—show a similar structural organization and signaling for nitrate [32,33,34]. Finally, ammonium is incorporated into C-skeletons in the form of glutamate by the glutamine synthetase/glutamate synthase (GS/GOGAT) cycle [35] (Figure 1).

It is important to point out here that in contrast to the low cytosolic concentrations of nitrite, those of nitrate are high (1–6 mM). Cytosolic nitrate levels are also more stably maintained than vacuolar concentrations (5–75 mM), considering that external nitrate concentrations may change by about 10,000-fold [36,37,38]. This is important for ensuring efficient nitrate assimilation, together with proper nitrate signaling in the tissues [38,39]. Nitrate homeostasis is the result of the membrane transporter-mediated supply of nitrate from vacuoles and the outer medium, as well as by nitrate efflux transporters such as NAXT1 [40]. NAXT1 belongs to the NRT1 family. In addition, the NR-catalyzed reaction has an effect, facilitating the conversion of nitrate into nitrite [37,38,41]. In the yeast *Hansenula polymorpha*, the sulfite transporter SSU2 and the nitrite transporter NAR1 have been characterized as essential components of the nitrate/nitrite efflux system [42].

## 3. Nitrate Reductase Is a Multidomain Protein

NR reduces nitrate to nitrite using electrons from NAD(P)H. The plant enzyme is about 200 KDa and contains two subunits, each bearing three prosthetic groups: FAD, heme b_557_, and molybdenum. In an NR subunit, molybdenum is bound to a tricyclic pyranopterin and chelated by a dithiolene, which is named the molybdenum cofactor (Moco). These domains are joined by two protease-sensitive hinge regions. The domains are redox centers, and electrons flow from NAD(P)H→FAD→ heme →Moco, which is within the active site for nitrate reduction [43,44]. Interestingly, the enzyme has two partial activities, which can be assayed in vitro: diaphorase, which catalyzes the reduction of artificial acceptors (ferricyanide or cytochrome c with NAD(P)H), and terminal-NR, which catalyzes nitrate reduction using electrons supplied by FAD, FMN, viologens, or bromophenol blue, chemically reduced by dithionite [43,44]. The crystal structure of the dimerized form of NR and Moco domains was solved [45].

## 4. Does NR Catalyze Nitrite Reduction to NO?

The experimental data have led to the proposal that the molybdoenzyme NR is the main enzyme responsible for NO production in most plants. This proposal was based on the experimental findings described below.

The first evidence linking NR and NO production was their co-elution by NADH from Blue Sepharose columns loaded with Soybean extracts with both NR and NO(X) evolution activities. In their main conclusion, the authors inferred their linkage from the fact that inhibiting the partial activities of NR and NO(X) evolution activities led to the same pattern. However, they went further by indicating that the terminal molybdenum-containing portion of NR is involved in the reduction of nitrite to NO(X) [46]. Other studies showed that several plant species emitted in vivo NO when there was nitrate in the soil, and the function was abolished in all plants in the study when they were grown on ammonium-containing soil, indicating a role for NR [47]. In addition, isotopically labeled ^15^N-nitrate resulted in the emission of ^15^NO [48]. Moreover, using NR from corn led to the production of significant amounts of NO from both nitrite and nitrate [49]. Those findings reinforced the idea that NR reduces nitrate to nitrite and further converts de novo-generated nitrite into NO. The proposal would be feasible if it could account for several facts. First, the nitrite-reducing activity of NR is very low (only 1% of the nitrate-reducing activity), and the Km of nitrite is about 10 times higher than that of nitrate, in agreement with the competitive character of nitrite (Ki = 50 μM) in nitrate reduction [50,51]. Taking into consideration the intracellular concentrations of nitrite (in the micromolar range) and nitrate (in the millimolar range), this nitrite-reducing activity would be irrelevant.

Another piece of evidence indicating the involvement of NR in NO production originated from the use of tungstate—an NR inhibitor—and NR-deficient mutants. In addition to its uncontrolled effects, such as metal toxicity, tungstate is a very unspecific inhibitor of Mo-enzymes. When exchanging Moco for the inactive tungsten cofactor (Wco) [52], all molybdoenzymes are inactivated, including mitochondrial amidoxime-reducing component (mARC), which is presented below as the most important enzyme in NO production under phototrophic conditions. Thus, tungstate is a clear inhibitor of NO production because of the resulting mARC inhibition and not because of NR inhibition. In agreement with this, Moco-deficient mutants are deficient in molybdoenzymes, as described in barley with a thermo-sensitive, wilty phenotype [53]. The most used Arabidopsis NR mutant to support the involvement of NR in NO production is the double mutant deficient in *NIA1* and *NIA2* genes. This nia1/nia2 double mutant has only 0.5% of the activity of wild-type NR and grows very poorly on medium with nitrate as the only nitrogen source [54]. As described below, NR is in fact involved in NO production but not through its Moco-dependent activity.

All five molybdoenzymes in plants (nitrate reductase, xanthine oxidase reductase (XOR), aldehyde oxidase (AO), sulfite oxidase (SO), and mARC) are able to catalyze the one-electron reduction of nitrite to NO. The molybdoenzymes are classified depending on how Moco binds to the enzyme’s active site: either covalently through an enzyme cysteine thiol group (NR, SO, and mARC) or with inorganic sulfur (XOR and AO) [55,56]. All of these enzymes show nitrite reductase activity to produce NO in vitro and in anaerobic conditions [44]. The four known human molybdenum-containing enzymes are the same as those in plants, except for NR, and they can also function as nitrite reductases under hypoxic conditions [56]. In mammals, two known pathways for NO formation are known: arginine oxidation under normoxic or aerobic conditions and nitrite reduction during hypoxia or anaerobiosis [56]. Plant SO seems to have a less potent nitrite reductase activity than human XOR and AO [57]. Whereas plant AO participates in the synthesis of phytohormones and contributes to reactive oxygen species (ROS) production, there is no information about its in vivo NO-producing activity [5]. 

Other processes that might produce NO from nitrite are associated with the plasma membrane-bound NR, which appears to be related to the mycorrhizal colonization of tobacco roots [58] and the mitochondrial electron transport chain (mETC), as demonstrated in several plants [59,60,61,62]. The mitochondrial complex III and IV are primarily implicated in the nitrite reaction (Km of 175 μM), which requires anaerobic conditions since oxygen is a strong inhibitor. Therefore, this reaction can occur in plant tissues exposed to hypoxia, such as roots, and its occurrence might be important to the plant by protecting the respiratory chain and mitochondrial metabolism when oxygen is lacking [63]. Recently, a role for Alternative Oxidase (AOX) in the production of large amounts of NO, observed under hypoxia, has been shown. In this pathway of NO production, AOX has a role in scavenging the NO and ROS linked to the hemoglobin–NO cycle, thus increasing energy efficiency without contributing to the formation of toxic peroxynitrite [64]. The implication of mitochondria in NO production from nitrite has also been shown in Chlamydomonas in the presence of high concentrations of nitrite [65].

In light of the biological importance of NO production, one would expect this process to be efficiently and finely regulated. Some of the Moco-dependent enzymes described above can mediate NO production under certain conditions, though it is difficult to currently envisage how to control this diversity of processes; even mitochondrial NO production requires anaerobiosis, suggesting a burst of NO synthesis from nitrite when this condition appears.

## 5. NR Does Not Catalyze In Vivo Nitrite Reduction to NO but Provides the Needed Electrons

Recently, NR was shown to be a necessary partner for NO production in a dual system, which, besides NR, includes another molybdoenzyme, mARC, since renamed NO-forming nitrite reductase (NOFNiR).

mARC has been extensively characterized in prokaryotic and eukaryotic organisms [66,67,68,69]. mARC proteins are about 35 KDa and require two electron transport proteins—NADH-cytochrome b5 reductase and cytochrome b5—to be functional. mARC is a very efficient reductase [70] for a number of N-oxygenated compounds, some of which are toxic or mutagenic [71,72,73]. This is why mARC has been related to cell detoxification processes. Both human mARC isoforms are associated with mitochondria, but mARC could also be located in peroxisomes because its two partners, NADH-cytochrome b5 reductase and cytochrome b5, were found in this organelle using a proteomic approach [74]. mARC was also connected to NO metabolism because of its activity on Nω-hydroxy-Nδ-methyl-L-arginine, an intermediate in the L-arginine-dependent biosynthesis of NO using NADH-cytochrome b5 reductase and cytochrome b5 [75]. In addition, human mARCs have nitric oxide synthase activity from nitrite with NADH and its two above-indicated partners [76].

In the eukaryotic alga Chlamydomonas, NO synthesis is carried out by a dual system comprising NR and NOFNiR (mARC). These two components are closely connected at both the transcriptional and activity levels, so mutants lacking one of them overexpress the other [77]. NR supplies NADH electrons to NOFNiR for the reduction of nitrite to NO much more efficiently than NADH-cytochrome b5 reductase and cytochrome b5. Both NR and NOFNiR are located in the cytosol [77].

NR mutants were found to be unable to provide electrons both in vitro and in vivo for NO synthesis. Some exceptions are NR mutants such as Chlamydomonas strain 301, whose NR lacks nitrate-reducing activity since its Moco domain is affected; however, it has functional diaphorase activity with intact FAD and heme domains [77]. So, the dual complex NR:NOFNiR produces NO independently of the molybdenum center of NR and depends on the NR electron transport chain from NAD(P)H to heme. 

NR:NOFNiR has been proposed to be the main system producing NO during standard phototrophic, oxygenic plant growth, which corresponds to most of its aerial part [77]. In tissues exposed to hypoxia, such as roots, other molybdoenzymes or mETCs could be involved in the synthesis of NO, as discussed above.

## 6. NO Levels in the Cells Are Regulated

The first line of control of NO levels is the regulation of its synthesis and degradation within the NO cycle (Figure 1). Members of the hemoglobin (HB) superfamily can oxygenate NO to nitrate, as was shown for Chlamydomonas THB1 [78], a class 3 truncated hemoglobin (THB) [79]. In different plant species, nitrate, nitrite, and NO upregulate HB expression [80,81]. In maize roots, the coordinated expression of both NR and HB also occurs [82]. Similarly, in Chlamydomonas, the expression of two truncated HBs, THB1 and THB2, respond selectively to N signals (nitrate, nitrite, and NO) and, interestingly, also to NIT2, the major regulatory gene of the nitrate assimilation pathway [78,83]. This regulatory gene is also essential for NR upregulation by nitrate [19]. The activity of THB1 requires electrons to be supplied by the NADH-diaphorase of NR, and the electron flow is likely from NADH to FAD [78]. Similarly, the activity of NOFNiR also requires the NADH-diaphorase of NR, but the electron flow is now from NADH to heme b [77]. Thus, nitrate through NIT2 would stimulate NO production because of NR’s increased expression (NOFNiR is not under NIT2 control), and in turn, NO degradation would occur as a result of stimulating both THB1 and NR. The homeostasis of NO is controlled by the activities of NR, NOFNiR, THB1, and THB2, which, in turn, depend on the relative concentrations of nitrate, nitrite, and NO, as well as NIT2.

So, just-synthesized NO, which is highly reactive, can react with different targets. Glutathione (γ-glutamylcysteinylglycine, GSH) is an essential metabolite in plants that participates in important functions, such as primary metabolism, redox signaling, and defense and detoxification processes [84]. 

GSH can react with NO to produce S-nitrosylated glutathione (GSNO). As a result, the half-life of NO in tissues available as a free radical gas changes from seconds to a few minutes. Thus, there are very sensitive mechanisms for regulating cellular processes. GSNO, which is considered the main reservoir for NO, provides NO signals to proteins. Protein S-nitrosation is considered the most important mode of action of NO. This is the covalent binding of NO to the thiol group of protein cysteine residues, and the creation of these bonds modifies the protein and can alter gene expression and/or lead to metabolic changes, all of which ultimately translate into physiological responses. Thousands of nitrosation sites have been identified in proteins [85]. GSNO is metabolized with GSNO reductase (GSNOR1) to convert it to glutathione disulfide (GSSG) and ammonia (NH3) [86,87]. GSNOR1 is a cytosolic enzyme that controls GSNO levels and, in this way, the nitrosation of proteins. GSNOR1 seems to be inhibited by NO, in which case, the scavenging of GSNO is prevented. Thus, NO controls its production and scavenging by regulating both nitrate assimilation efficiency and GSNOR1 activity [87,88] (Figure 1).

Other posttranslational modifications of proteins mediated by NO are the nitration of tyrosine and amine groups and the oxidation of thiols and tyrosine [89]. NO can react with superoxide (O_2_^−^) to form peroxinitrite (ONOO^−^), which is a powerful oxidant contributing to the nitration of protein tyrosine residues to form 3-nitrotyrosine, which results in regulatory responses. Nitration seems to be a reversible process that might occur at specific tyrosine residues depending on the local environment and the secondary and tertiary structure of the protein. A putative specific denitrase removes the nitro group without degrading the protein, as has been shown in animal systems [89,90].

Reactive oxygen species (ROS), such as oxygen, singlet oxygen, hydroxyl radical, hydrogen peroxide, and superoxide anion, all of which are important signaling compounds produced under several environmental conditions, interact with NO and other reactive nitrogen species (RNS). They mediate the responses to different environmental situations, even promoting the systemic adaptation of plants to stress situations [2,91,92].

## 7. The NO Synthesis Systems Are Coordinated with Nitrogen Metabolism

Under circumstances in which NR is not present or active, another NO-forming system accounts for NO production and NO effects. Analysis of this topic and possible future directions are presented here.

With an ammonium medium as the sole N-source or in null NR-deficient mutants, NR is absent, and thus, the dual system NR–NOFNiR is not functional. Nevertheless, NO is being synthesized, probably due to the existence of a NOS-like activity in algae and plants. This activity can be inhibited by some compounds, which are primarily arginine analogs acting on the animal-type NOS [6,17].

Plant peroxisomes are single-membrane-bound organelles with an oxidative metabolism and a simple morphology, but they also have a complex composition of enzymes involved in the metabolism of oxygen free-radicals. Peroxisomes can generate ROS and nitric oxide and thus important signal molecules with implications for cellular metabolism in plants [91,93].

Many studies of different plant species have shown the presence of L-Arg-dependent NO synthase-like enzyme activity, which has biochemical requirements similar to animal NOS (L-Arg, NADPH, FMN, FAD, Calmodulin, and Ca^2+^) [94,95]. Corpas and Barroso (2017) postulated that the L-Arg-dependent NO synthesis that occurs in plants could correspond to cooperation among discrete proteins, resulting in the formation of a protein complex with requirements for enzyme activity that are similar to animal NOS [96]. This would explain the lack of success in finding canonical NOS proteins at the molecular level.

On the other hand, coordination between nitrogen assimilation and the nutritional status within plant metabolism is a critical issue for plant viability. A very abundant family of N- and C-signaling proteins, widely distributed in nature, is the PII protein family from bacteria, archaea, and plants [97]. In plants, these nuclear-encoded PII proteins localize in the chloroplast and are not subject to the covalent modification reported for bacterial PII [98]. PII senses and integrates the signals of the C–N balance in the cells using 2-ketoglutarate as an indicator, together with the energy status by competitive ADP/ATP binding [99,100]. Plant PII proteins are conserved throughout the evolutionary history of the Chloroplastida—green algae and land plants [97]—and participate in a complex signal-transduction network that mediates nitrogen regulation [101]. In Arabidopsis, the PII protein controls arginine biosynthesis [102], and PII mutants show an enhanced nitrite uptake by the chloroplast [103]. By binding effector molecules, PII interacts with and signals to other chloroplast proteins, such as N-acetyl-L-glutamate kinase (NAGK), which catalyzes the committed step in the arginine biosynthesis pathway [102] (Figure 1). Among the effectors binding plant PII protein is glutamine, which was shown to bind to the termed Q-loop of the C-terminus crystalized plant PII protein [104]. The binding of glutamine to PII changes its conformation to one that interacts and activates NAGK for the synthesis of arginine and polyamines. PII–glutamine sensing is a widespread mechanism in the plant kingdom [104]. In Chlamydomonas, arginine is a component of the same repressive pathway as ammonium and CYG56, which is a guanylate cyclase activated by NO and needed to repress nitrogen assimilation genes [105].

Interestingly, plant PII seems to be connected to the regulation of nitrite metabolism, so PII knockout mutants show an increased sensitivity to nitrite and a decrease in total amino acids, especially glutamine. Lack of PII seems to increase the C/N balance, as previously shown in cyanobacteria [30]. Recently, Chlamydomonas PII levels have been shown to be tightly controlled by the nitrogen source and the physiological status of the cells [106]. In fact, PII expression is subject to positive (nitrate and nitrite) signaling and is downregulated by ammonium via an NO-mediated process that involves an NO-dependent guanylate cyclase, similar to the negative effect of ammonium on NR expression [6]. PII expression is very similar to that of nitrate assimilation genes [25], so an interaction/coordination between PII and nitrate assimilation pathways has been suggested [106].

Under standard phototropic conditions, nitrate assimilation takes place under the positive control of nitrate, mediated by the regulatory gene *NIT2* [25]. When both nitrate and ammonium are present, there is a balance between the positive and negative signals, and NR expression follows that balance; the NR transcript is detectable even in the presence of ammonium, provided that nitrate is also present [107]. The negative signal of ammonium on Chlamydomonas NIA1 gene expression depends on NO and its mediation by an NO-dependent guanylate cyclase (CYG56). So, NO concentrations in the cells increase with ammonium concentration, leading to complete NR repression [107] by means of a mechanism that is dependent on a possible NOS, which is inhibited by L-NAME.

In different plants, NO production is also sensitive to the mammalian NO synthase inhibitor L-NAME [2,5]. L-NAME was also shown to affect NO production by interfering with NR activity [108], which seems to question the mechanisms of action of this compound. However, it has to be considered that in the double nia1/nia2 Arabidopsis mutant commonly used to study NO effects [54], the content of nitrite, as expected from the NR deficiency, and of free amino acids, particularly L-arginine, are much lower than in wild-type plants [109]. Thus, both substrates for NO synthesis would be compromised. 

In conclusion, PII proteins mediate the signaling of the N-source with respect to the carbon status (2-ketoglutarate). This N can be either oxidized (nitrate, nitrite) with a generally high C/N balance, or it can be reduced (ammonium) with a generally low C/N balance. With a high C/N balance, the nitrate assimilation pathway is operative, and NR expression would favor the increase in N capture and its incorporation into C-skeletons (2-ketoglutarate), which would be abundant. When the C/N balance is low, NR would be repressed, and the biosynthesis of arginine would be stimulated. So, under these two extreme conditions, the substrates to produce NO would change. In conditions of high C/N, nitrite would be efficiently produced; however, with low C/N, arginine biosynthesis would predominate with low nitrite production. So, it is proposed that the dual NR:NOFNiR system will preponderate at high C/N, whereas the NOS-like system will be mostly operative at low C/N. PII protein expression will follow a pattern similar to that of NR [110] to enhance N acquisition and balance the C/N ratio. These two extreme situations might be changed to intermediate ones, depending on the C/N balance of the cells.

## 8. Conclusions

Nitric oxide is such an important signaling molecule that its production and scavenging must be tightly regulated. Some of the biosynthetic mechanisms are starting to be disentangled, while others still require additional useful information for their elucidation. Part of the confusion regarding the primary source of NO might come from the fact that several pathways might function simultaneously to different extents, depending on nutritional and environmental conditions. Many points still have to be clarified for plants.
